# *Paraphysoderma sedebokerense* Infection in Three Economically Valuable Microalgae: Host Preference Correlates with Parasite Fitness

**DOI:** 10.3390/jof7020100

**Published:** 2021-02-01

**Authors:** David Alors, Sammy Boussiba, Aliza Zarka

**Affiliations:** Microalgal Biotechnology Laboratory, the Jacob Blaustein Institutes for Desert Research, Ben Gurion University of the Negev, Sede-Boker Campus 8499000, Israel; sammy@bgu.ac.il

**Keywords:** *Paraphysoderma sedebokerense*, *Haematococcus pluvialis*, *Chromochloris zofingiensis*, *Scenedesmus dimorphus*, parasite fitness, prevalence, intensity of infection

## Abstract

The blastocladialean fungus *Paraphysoderma sedebokerense* parasitizes three microalgae species of economic interest: *Haematococcus pluvialis*, *Chromochloris zofingiensis* and *Scenedesmus dimorphus*. For the first time, we characterized the developmental stages of isolated fungal propagules in *H. pluvialis* co-culture, finding a generation time of 16 h. We established a patho-system to compare the infection in the three different host species for 48 h, with two different setups to quantify parameters of the infection and parameters of the parasite fitness. The prevalence of the parasite in *H. pluvialis* and *C. zofingiensis* cultures was 100%, but only 20% in *S. dimorphus* culture. The infection of *S. dimorphus* not only reached lower prevalence but was also qualitatively different; the infection developed preferentially on senescent cells and more resting cysts were produced, being consistent with a reservoir host. In addition, we carried out cross infection experiments and the inoculation of a mixed algal culture containing the three microalgae, to determine the susceptibility of the host species and to investigate the preference of *P. sedebokerense* for these microalgae. The three tested microalgae showed different susceptibility to *P. sedebokerense*, which correlates with blastoclad’s preference to the host in the following order: *H. pluvialis* > *C. zofingiensis* > *S. dimorphus*.

## 1. Introduction

The aquatic lower fungi belonging to Blastocladiomycota [1] and their close relatives, the better studied Chytridiomycota, have a high impact on phytoplankton dynamics [2,3], and commonly constitute a plague for microalgae in mass outdoor cultures [4,5,6]. However, little is known about the life cycle, ecology and host range of many algal parasites [7]. Even more, the time needed to complete the life cycle of parasitic Chytridiomycota and Blastocladiomycota species, an essential parameter for understanding epidemics, has not been investigated and is only rarely reported from in situ studies [8,9,10,11,12].

*Paraphysoderma sedebokerense* is a facultative parasite, which was isolated for the first time from *Haematococcus pluvialis* collapsed cultures in Israel and can be maintained in laboratory culture growing as a saprobe [13]. The life cycle of *P. sedebokerense* is complex and includes both the thin cell wall vegetative cysts, and the darker resting cysts with thicker cell wall which acts as a resistant stage, and two types of dispersion propagules: flagellated zoospores (produced only in infected cultures) and amoeboid swarmers [13,14]. In the infective stage, the propagules cause lethal epidemics in *H. pluvialis*, which is the best natural source of the high value ketocarotenoid astaxanthin [15]. The economic impact of these epidemics is reflected in efforts to control the development of the infection by *P. sedebokerense*, by selection of resistant *H. pluvialis* strains phenotypically dominated by motile cells which are not vulnerable to the parasite [16,17], changing culture conditions [18] or using surfactants [19]. In laboratory tests, *P. sedebokerense* showed the ability to also infect other microalgae; *Chromocloris zofingiensis* could be infected—at least under some conditions—to the same extent as *H. pluvialis*, and *Scendesmus vacuolatus* was also parasitized, albeit with low infectivity [20]. Recently, *P. sedebokerense* was also isolated from an outdoor collapsed culture of *Scenedesmus dimorphus* in the United States [21]. The infection of *C. zofingiensis* and *S. dimorphus* by *P. sedebokerense* could have economic importance, since the algal hosts are sources of antioxidant substances and lipids with applications in pharmacological, nutraceutical and biofuel energy industries [22,23].

The closest genus to *Paraphysoderma* is the genus *Physoderma*, that parasitizes species of higher plants [13,24]; both genera altogether are the only Blastocladiomycota that parasitize the kingdom plantae [24]. In contrast to *Paraphysoderma*, which is a recently identified genus with only one species that can infect unrelated algal hosts, *Physoderma* comprises 99 species [25] of presumably species-specific parasites identified from the year 1833 to the year 1977, which are named with the epithet of the plant host from which they were isolated. As far as we know, this specificity of *Physoderma* species was poorly tested, and mainly it was assumed [26]. In phytoplankton parasites, different host specificity was reported when host-range was investigated “in situ” or “in vitro”, commonly showing host specialist parasites in field studies [2,3,27] and host-generalist parasites in laboratory studies [6,28,29,30]. These differences suggest that the “in situ” studies show the preferred host suite, while “in vitro” studies show all the potential hosts [31]. This ecological pattern has been described as an evolutionary trend in parasites and allows specific parasites to become generalist at a higher frequency than was previously expected [32,33,34,35]; this also occurred in fungal evolution [36,37,38]. As a first step of adaption to a new host, some parasites of microalgae can infect suboptimal hosts but produce offspring with decreased infectious capability [39].

Recently, a method to stimulate the synchronous production and isolation of purified propagules, from both pure *P. sedebokerense* and infected *H. pluvialis* culture, has been developed in our laboratory [40]. Here, we took advantage of this method to establish a system for quantitative infection tests of *H. pluvialis* by *P. sedebokerense* propagules, to elucidate the infection process by describing the kinetics of a complete infection cycle ending with the generation of new propagules. With this established *H. pluvialis* infection system, we answer the question of preference of the blastoclad for the different algal species previously described as suitable hosts, and compare the kinetics of the infection and the fitness of the parasite in these three different microalgae (i.e., *H. pluvialis*, *C. zofingiensis* and *S. dimormphus*). We show that *P. sedebokerense* preference is strongly coupled with reproductive fitness preferring the microalgae that offers the best source of nutrients. We also compared quantitatively and qualitatively the progress of the infection in three highly valuable microalgae.

## 2. Materials and Methods

### 2.1. Blastoclad Culture

We used the blastoclad *Paraphysoderma sedebokerense* isolate AZ_ISR (2019), considering it is the same TJ-2007a strain described in Hoffman et al. [13], since it was isolated from the same host (*H. pluvialis*) and from the same place at Sede-Boker, Israel. The ITS sequence (GenBank MW336992) of our isolate is 99.7% identical to *P. sedebokerense* isolates FD61 [21] and JEL0821 [41]. After isolation, we maintained it in a pure culture and continuously subculture it on solid blastoclad growth media (BGM) in our laboratory [40]. Pure cultures of blastoclad were grown in BGM at 30 °C in an incubator shaker (180 rpm) supplemented with 2% CO_2_, under continuous dim white light (15 μmol photons m^−2^ s^−1^) illumination [40]. Blastoclad propagules were harvested in propagules stimulation medium (PSM), following the protocol developed by Asatryan et al. [40]. The quality of the propagules was visually checked under light microscopy, with attention to propagule movement, absence of encysted propagules and general appearance. Propagules were directly counted in a Neubauer chamber (Reichert Bright-Line 1492 hemacytometer, Hausser Scientific, Horsham, PA, USA), at 400× magnification with phase contrast, using a Zeiss AxioSkop HBO 50 W microscope (Zeiss, Oberkochen, Germany).

### 2.2. Algal Strains and Growth Conditions

*Haematococcus pluvialis* Flotow 1844 em. Wille K-0084 was obtained from Scandinavia Culture Center for Algae and Protozoa at the University of Copenhagen, Denmark. *Scenedesmus dimorphus* UTEX B 1237 was obtained from the culture collection of algae at the University of Texas at Austin. *Chromochloris zofingiensis* SAG-211.14 was obtained from the Culture Collection of Algae of the University of Göttingen (SAG), Göttingen, Germany. Algal monocultures were grown for 7 days in modified BG-11 (mBG11 [42]), in 250 mL Erlenmeyer flasks (100 mL culture), at controlled temperature (25 °C), and constant illumination (80 μmol photon m^−2^ s^−1^), in an incubator shaker (150 rpm) enriched with 2% CO_2_. For maintenance, *H. pluvialis* cultures were weekly diluted to approximate densities of 2 × 10^5^ cells/mL. Maintenance of *C. zofingiensis* and *S. dimorphus* cultures was also done by continuous dilutions of cultures to approximately 5 × 10^6^ cells/mL. At these cell densities, cultures reach the stationary stage after one week.

### 2.3. Estimation of Algal Cell Surface Area

Using a LUNA-FL™ Dual Fluorescence Cell Counter automated cell counter (Logos Biosystems, Gyunggi-do, Korea), we measured the cell diameter (long axis for Sd) of each species used in this work. For *H. pluvialis* and *C. zofingiensis*, we considered them as perfect spheres to calculate their surface area (4 × π × r^2^). For *S. dimorphus*, we measured the two axes, and we simplified the calculation, considering S. dimorphus cells as cylinders without base, to calculate the surface area (2 × r × π × h) (Supplementary Table S1).

### 2.4. Infection of Algal Cultures and Infection Parameters

The infections were carried out in fresh mBG11 medium, in the same conditions used to grow the blastoclad, as described above. We tested different types of inoculum: *P. sedebokerense* logarithmic monoculture, blastoclad coculture with *H. pluvialis* (Ps-Hp) and purified propagules routinely isolated from 7–10 days old blastoclad monoculture (referred to in the text as pure propagules) or in specific experiments purified propagules isolated from blastoclad coculture with *H. pluvialis* 3 days after inoculation (referred to as propagules isolated from Ps-Hp coculture).

Macroscopically, we assessed the virulence of the blastoclad against different algal monocultures as the time needed to cause collapse of the algal culture (flocculation and color change from green to brown), using all different types of inoculums described above.

The quantitative infections were all done only with *P. sedebokerense* pure propagules, isolated from blastoclad monoculture. To avoid bias, we compared the infection development in the three microalgae (*H. pluvialis* 4 × 10^5^ cells/mL, *C. zofingiensis* 4 × 10^6^ cells/mL, and *S. dimorphus* 1 × 10^6^ cells/mL, cell densities which offer the same host surface area per mL) by inoculating synchronously with the same batch of fresh pure propagules. We assessed five parameters: prevalence (Pr.) as the percentage of host cells carrying encysted parasite, the average number of parasites cysts per cell (considering all cells) as intensity (In.), the average number of parasites cysts per algal surface area as areal density (Ad.), the propagule survival as the sum of host attached cysts and free-swimming propagules at the indicated time (before the first propagule release) out of the total inoculated propagules at zero time of the experiment and propagule production as the measured number of propagules released from the inoculated cultures. The values of Ad. are in areal units; in our case, a unit area is the average surface area of a *C. zofingiensis* cell (88 µm^2^, Supplementary Table S1).

Since we used different densities of microalgae normalized on their surface area to have the same probability for the parasite to find a suitable host, we needed to use different inoculation ratios. One ratio to quantify the parameters of the infection which are related to the ratio of parasite per host, choosing 1:1 to allow fast development of the infection without excessive effort to harvest propagules. For the parameters of the fitness, we used the same number of propagules per algal culture, to compare survival and production with respect to the inoculated amount. Since we fixed different microalgal cells densities and we needed the same infective ratio for infection parameters and the same number of propagules inoculated for fitness parameters, it ended up with different ratios for each setup.

We considered Pr., In., and Ad. as parameters of infection, and to measure these parameters, we inoculated *P. sedebokerense* propagules/host cell at the same ratio (1:1). To detect the encysted propagules, we used the combination of phase contrast and fluorescent Nile red staining of the blastoclad [20], at 40× magnifications when inoculated with *H. pluvialis* and *S. dimorphus* cultures, and at 100× magnifications when inoculated with *C. zofingiensis* cultures. For quantification, 50 host cells were counted and considered as a representative subsample.

We considered propagule survival and propagule production as fitness parameters of the parasite, and we inoculated the same number of propagules per infected culture resulting in dissimilar ratios of infection (10:1 in *H. pluvialis*, 4:1 in *S. dimorphus* and 1:1 in *C. zofingiensis*) and determine both parameters by direct cell counting under the microscope. To measure propagule survival, we counted both free-swimming propagules and encysted ones, considering as live propagules those keeping the amoeboid shape and dead propagules the burst or collapsed ones (which were not counted). Since we inoculate a known number of propagules, we calculated the percentage of survival as = (counted number of live propagules and cysts at each time point/number of inoculated propagules at time zero) × 100.

For each parameter, at each sampling point and microalgal species, three to five replicates were measured.

Finally, we considered preference as the successful encystment events of the blastoclad propagules on a specific microalgal species when the other microalgae species are present. We assessed the preference by inoculating purified propagules in a mixed algal culture, where each of the three algae were present at a density that offered equal surface area to the parasite, hereafter referred to as “mixed culture”: *H. pluvialis* at 2.5 × 10^4^ cells/mL, *S. dimorphus* at 7.5 × 10^4^ cells/mL and *C. zofingiensis* at 2.1 × 10^5^ cells/mL. The preference was calculated as the number of encystment events divided by the maximum possible encystment events (under assumption that all propagules were alive at the onset of inoculation), multiplied by 100.

Preference = (In. microalga × microalga cell number) / (number of inoculated propagules).

For additional details about the inoculum type and conditions of the experiments, see supplementary material (Supplementary Table S2)

### 2.5. Statistical Analyses

Statistical analyses were performed in StatGraphics centurion XVII, considering statistically significant p-values lower than 0.05. The comparison of infection parameters and the fitness parameters of parasite of algal cocultures was done by general linearized model (GLM), separately for each sampling time. To discriminate which means are different, a multiple comparison was performed using the Fisher method (Supplementary Table S3).

The statistical analysis for the preference test was manually done using the chi-square:

x^2^ = ((Observed-Expected)^2^)/Expected and comparing the value of the results with the chi-square tables.

## 3. Results

### 3.1. Infection Cycle of H. pluvialis by P. sedebokerense Isolated Pure Propagules

In order to establish the infection system, the chronology of the infection of *H. pluvialis* by *P. sedebokerense* was assessed via inoculating pure amoeboid swarmers of *P. sedebokerense* (obtained from pure culture in BGM) (Figure S1) and following the different developmental stages during the first infection cycle, until new propagules are released.

**Figure 1:**
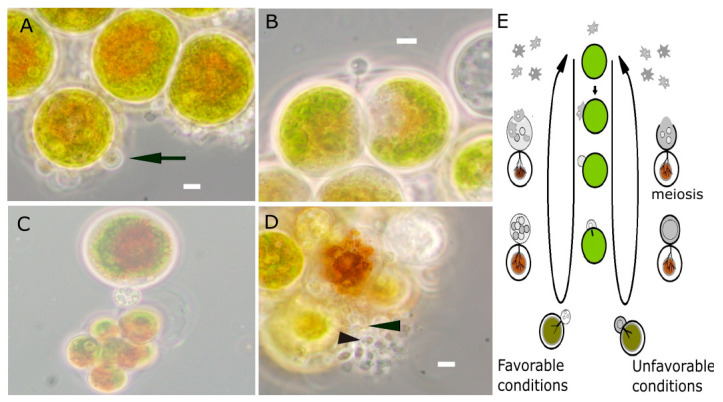
Phase contrast images of the first infection cycle of *H. pluvialis* by *P. sedebokerense* pure propagules and a schematic representation modified from Hoffman et al. 2008. (**A**) At 0.5 h after inoculation, the propagules are round as they start to encyst (arrow). (**B**) At 1 h after inoculation, the cysts produce a germ tube which penetrates the host cell wall. (**C**) At 9 h after inoculation, the cysts mature and lipid droplet clusters suggest that cyst turned into sporangium. (**D**) Moreover, 16 h after inoculation, the discharge pore (arrowhead) of the sporangia opened and the new next generation of amoeboid swarmers are released. Scale bars: 5 µm. (**E**) Schematic representation of the life cycle of the infection of *P. sedebokerense* infecting *H. pluvialis*, showing the progression of the cycle under favorable and unfavorable conditions with formation of resting cysts with thicker cell wall and characteristic ventral vacuole that disappears prior to germinating.

The attachment of the propagules to the algal cells that begins the encystment process is visible 30 min after inoculation, when the shape of the amoeboid swarmers changes into a completely round cyst-like structure (Figure 1A). One hour after inoculation, the encysted propagule penetrates the algal cell wall with a germ tube (Figure 1B) and the rhizoidal system grows. After encystment, the blastoclad cyst feeds on the algal cell and consequently grows in size; it initiates cell division 9 h after inoculation (Figure 1C). The mature sporangium, which loses its spherical shape when propagules increase the pressure on its cell wall, is visible 16 h after inoculation. At this stage, the discharge pore of the sporangia opens to enable the release of new infective propagules, completing the first infection cycle (Figure 1D).

### 3.2. Virulence of P. sedebokerense against Different Algae

**Figure 2:**
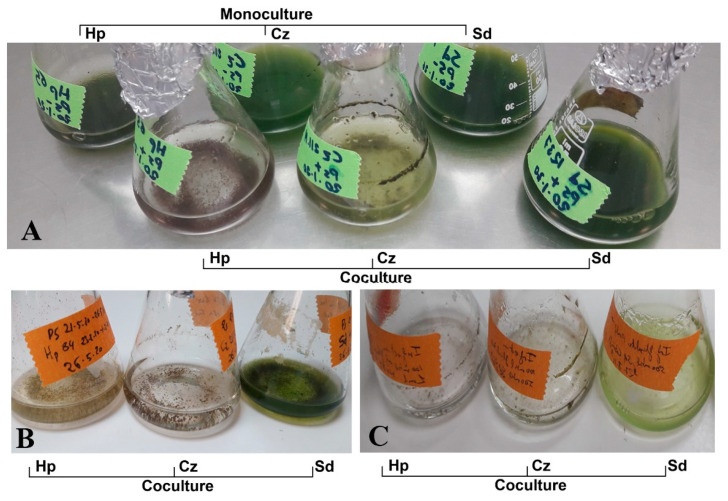
Virulence of different types of *P. sedebokerense* inoculum against *H. pluvialis* (Hp), *C. zofingiensis* (Cz) and *S. dimorphus* (Sd) algal cultures. (**A**) Algal monocultures inoculated with logarithmic *P. sedebokerense* cultures, 5 days after inoculation and negative controls (the same algal monocultures without inoculation of the parasite). (**B**) Algal monocultures 2 weeks after inoculation with Ps-Hp coculture (same volume of inoculum to each algal culture). (**C**) Algal monocultures 5 days after inoculation with isolated propagules from Ps-Hp coculture, containing both amoeboid swarmers and flagellated zoospores. (ratio infection Ps-Hp 1:3; Ps-Cz 1:30, P-Sd 1:10).

**Figure 3:**
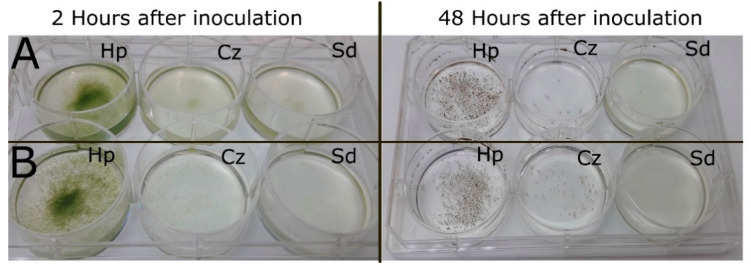
Virulence of purified propagules isolated from *P. sedebokerense* culture 2 and 48 h after inoculation. (**A**) Algal monocultures inoculated at a propagule/host cell ratio of 1:1 to allow comparisons of prevalence and intensity of infection between microalgal species. (**B**) Algal monocultures inoculated with the same number of propagules at ratios of 10:1 Ps-Hp, 4:1 Ps-Sd and 1:1 Ps-Cz, adjusted to show similar algal surface area, to allow comparison of propagule survival and propagule production between microalgal species. Hp: *H. pluvialis*, Cz: *C. zofingiensis* and Sd: *S. dimorphus*.

We inoculated *H. pluvialis*, *S. dimorphus* and *C. zofingiensis* with *P. sedebokerense*, assessing the virulence macroscopically, by observing the flocculation and change of color of the algal cultures from green to brown (Figure 2 and Figure 3); the macroscopic observations were confirmed under a light microscope. For inoculation, *P. sedebokerense* logarithmic cultures (Figure 2A), previously infected *H. pluvialis* monocultures (Figure 2B), or propagules isolated from Ps-Hp coculture (Figure 2C) or pure isolated propagules (Figure 3) were all tested; these four inoculum types showed similar virulence, but the susceptibility of the three different microalgae was different. *H. pluvialis* showed high susceptibility to the infection, collapsing in 24–72 h after inoculation, depending on the amount of *P. sedebokerense* inoculum. We found a similarly high virulence of *P. sedebokerense* in *C. zofingiensis* cultures, which collapsed 24–96 h after inoculation in most of the cases. In contrast, we never observed total collapse of the *S. dimorphus* culture after inoculation with *P. sedebokerense*; the culture always maintained its vivid green color, although partial flocculation of the culture was observed (Figure 2B). These symptoms in *S. dimorphus* cultures never progressed, even if we used higher *P. sedebokerense* inoculum and/or if we maintained the inoculated culture for longer time (at least two weeks). Only for *S. dimorphus*, infection tests were also conducted in the medium used by Letcher et al. [21], and still collapse of the culture was not observed.

### 3.3. Infection in Mixed Algal Culture and Cross Infections

A mixed algal culture composed of *H. pluvialis* (2.5 × 10^4^ cell/mL), *S. dimorphus* (7.5 × 10^4^ cell/mL) and *C. zofingiensis* (2.1 × 10^5^ cell/mL) was prepared and inoculated with blastoclad pure propagules (2.3 × 10^5^ propagules/mL). These algal densities were chosen in order to expose the same surface area of each algal species to the blastoclad inoculum. We measured the preference as the percentage of inoculated propagules that successfully encysted on live green algae. We expect the same number of encystment events (7.67 × 10^4^) for each of the three microalgae, if the blastoclad has no preference for a specific algal specie. However, 9 h after inoculation, 52.1% of the propagules encysted on *H. pluvialis* cells, 1.6% of propagules encysted on *C. zofingiensis* cells, while no cysts were detected on *S. dimorphus* (Table 1). The differences between observed and expected encystment events were statistically significant under chi-square test with a pValue lower than 0.001. We thus conclude that *P. sedebokerense* shows high preference for *H. pluvialis* cells, intermediate preference for *C. zofingiensis* and null preference for *S. dimorphus*. Moreover, 20 h after inoculation all *H. pluvialis* cells carried *P. sedebokerense* cysts, while 42% of *C. zofingiensis* cells and 95% of *S. dimorphus* cells remained uninfected and parasite-free 48 h after inoculation (data not shown). A big proportion of the inoculated propagules were not directly attached to any of the three microalgae; it was not quantified, but many of them encysted on empty mother cell walls (especially from *H. pluvialis*) and on other blastoclad cysts.

To test cross infections, fresh algal monocultures were inoculated with the five-days-old infected algal monocultures described in Figure 2C. The results showed different susceptibility to the blastoclad, depending not only on the algal host, but also depending on the type of inoculum (Table 2). *H. pluvialis* was the most sensitive monoculture, suffering fast culture collapse 48–60 h after inoculation with all four inoculum types; at the same time, Ps-Hp co-culture was the most virulent towards both *H. pluvialis* and *C. zofingiensis*. The *C. zofingiensis* monoculture showed less susceptibility as compared to *H. pluvialis* monoculture; epidemics developed slower and cultures collapsed 60–168 h after inoculation, depending on the inoculum type Table 2. The inocula of Ps-Cz and Ps-Sd co-cultures showed similar virulence towards *C. zofingiensis*, causing only flocculation and lower virulence than Ps-Hp and Ps-mixed co-cultures, which caused culture collapse in *C. zofingiensis* (Table 2) In all tests, *S. dimorphus* did not collapse nor flocculate.

### 3.4. Quantification of the Infection

**Figure 4:**
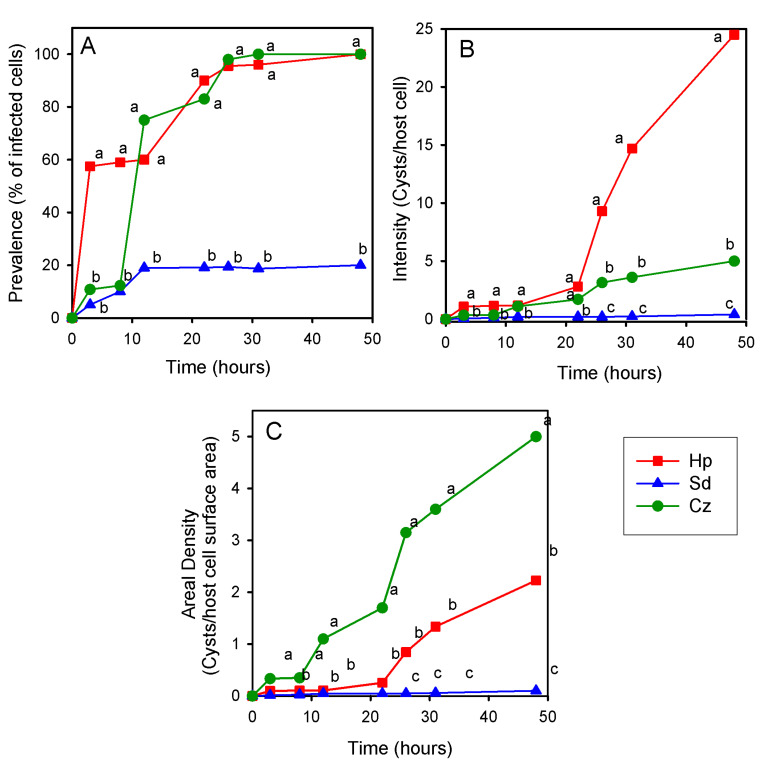
Infection parameters in co-cultures of *H. pluvialis* (Hp), *C. zofingiensis* (Cz) and *S. dimorphus* (Sd) with *P. sedebokerense* pure propagules inoculated at 1:1 ratio of propagules/host cell, during the first 48 h after inoculation. (**A**) Prevalence, (**B**) Intensity (considering infected and non-infected cells) and (**C**) Areal density per unit area were measured. We normalized the unit areas, considering 1-unit area is the average cell surface of *C. zofingiensis* (for more details, see Supplementary Table S1). Data presented in this figure are from cultures shown in Figure 3A. Letters indicate statistical significance (*p* value < 0.05).

The cell size is remarkably different between the tested algal species. To offer the parasite the same probability to encyst on an algal cell, we used cell densities which provide equal surface area per ml in each of the tests; consequently, algal cell density is different for each tested alga. To enable the comparable measurement of the prevalence (% of infected host cells) and intensity (average number of cyst/host) of the infection (parameters that are expressed on the basis of individual algal host cell), the three different microalgae were inoculated with *P. sedebokerense* pure amoeboid swarmers at a ratio of 1:1 (parasite/host). We found statistical differences in prevalence values (α 0.05); *H. pluvialis* suffered higher prevalence than *C. zofingiensis* in the first 8 h and higher than *S. dimorphus* during the 48 h of the experiment. During the first day, at sampling points 3, 8 and 12 h, *H. pluvialis* showed high and constant prevalence of about 60%; the prevalence increased, reaching 100% of prevalence 24 h after inoculation (Figure 4A). In the case of *C. zofingiensis*, the initial prevalence (3 h and 8 h after inoculation) was low (<12%) and not statistically different from that of *S. dimorphus* (Supplementary Table S3). However, the prevalence sharply increased later on, to 75% at 12 h after inoculation. In contrast, the prevalence in *S. dimorphus* increased slowly, reaching a maximum of 20%, 12 h after inoculation, and stayed the same until the end of the experiment (48 h) (Figure 4A).

We also found statistical differences (α 0.05) in the intensity of the infection in the three microalgae: *H. pluvialis* suffered higher intensity of the infection as compared to the other two microalgae, and only at 12 h and 22 h after inoculation, the intensity in *H. pluvialis* is not statistically different from *C. zofingiensis* intensity. The intensity of the infection in *S. dimorphus* was the lowest, and only in the first sampling point (3 h) it was not statistically lower than the intensity of the infection in *C. zofingiensis* (Supplementary Table S3). At the end of the experiment (48 h after inoculation), the intensity of the infection (cyst/host cell) reached values of 25, 5 in *H. pluvialis* and *C. zofingiensis*, respectively, and 0.4 in *S. dimorphus* (if only infected cells are being considered) (Figure 4B). Interestingly, if we express the intensity of infection on the basis of a unit of surface area (considering the average surface area of *C. zofingiensis*, the smallest alga as one unit), the areal density is the highest in *C. zofingiensis*. The areal density of the infection in *C. zofingiensis* was significantly different from that of *H. pluvialis* and *S. dimorphus*, which were significantly different from each other only after 22 h after inoculation. At the end of the experiment (48 h) *C. zofingiensis*, *H. pluvialis*, and *S. dimorphus* carried 5, 2.2, and 0.1 cysts per unit area, respectively (Figure 4C). We noticed a similar pattern of changes in prevalence values of *H. pluvialis* and *C. zofingiensis*, during the first 22 h after inoculation. In *H. pluvialis*, prevalence sharply increased at time intervals of 0–3 and 12–20 h, while in *C. zofingiensis*, it increased at time intervals of 0–3, 8–12 and 20–22 h after inoculation (Figure 4A). In both algae, the first increase phase is due to the attachment/encystment of the inoculated propagules, while the other increase phases are suggesting new propagule release. The consequent increases in intensity and areal density are in accordance with the propagule release pulses. In the case of *S. dimorphus*, a plateau was reached 12 h after inoculation, with no further increase (Figure 4C) within the two days of the experiment.

**Figure 5:**
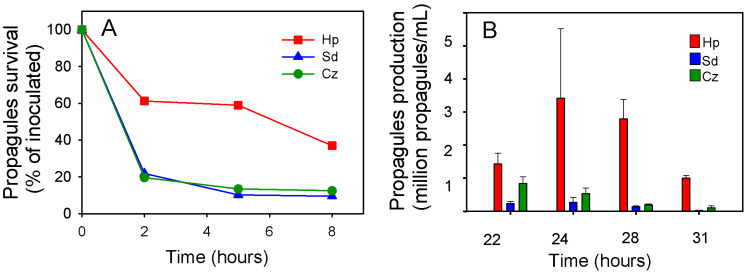
Fitness parameters of the parasite in coculture of *H. pluvialis* (Hp), *C. zofingiensis* (Cz) and *S. dimorphus* (Sd) inoculated with same number of purified propagules. (**A**) Propagule survival measured in the first hours after inoculation as the sum of free-swimming propagules and encysted ones; (**B**) Propagule production counted in hemacytometer. The same cultures were used to assess propagule survival and propagule production, and in each coculture, the same number of propagules was inoculated in different microalgal densities adjusted to offer the same algal surface area. Data presented in this figure are from cultures shown in Figure 3B. Letters indicate statistical significance (*p* value < 0.05).

Blastoclad fitness tests were conducted as mentioned above with inoculations of algal monocultures based on equal algal cell surface area. However, in this experiment, inoculation with the blastoclad was done with an equal propagule number in each tested alga culture, to enable the comparison of survival and production with respect of the inoculated amount at time zero. Here, we found higher propagule survival in *H. pluvialis* (*p* value 0.047) than in *C. zofingiensis* and *S. dimorphus* monocultures (Figure 5A). Propagule production was different among the different algae. The values in *H. pluvialis* were significantly different from the other algae in each sampling time (*p* value 0.0054), while the production in *C. zofingiensis* was different from *S. dimorphus* only at the measured peak of production (22 h after inoculation). In *H. pluvialis*, a peak of 3.4 × 10^6^ propagules/mL was observed after 24 h, in *C. zofingiensis* it was observed after 22 h (8.4 × 10^5^ propagules/mL), and in *S. dimorphus* it was observed 24 h after inoculation (2.7 × 10^5^ propagules/mL) (Figure 5B).

**Figure 6:**
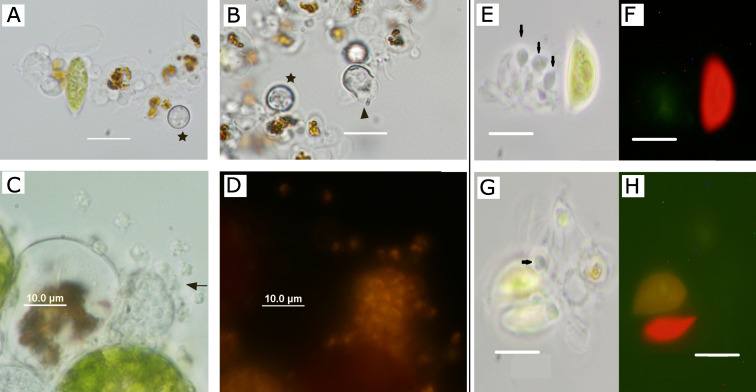
Comparison of *S. dimorphus* inoculated cultures (**A**,**B**,**E**–**H**) and *H. pluvialis* propagules release (**C**,**D**). (**A**) *S. dimorphus* infected cells carrying *P. sedebokerense* cysts; thicker cell wall resting cyst is marked by a star, 27 h after inoculation; (**B**) Resting cyst of *P. sedebokerense* (arrowhead) germinating and liberating the sporangium to media 48 h after inoculation; (**C**,**D**) vegetative sporangium of *P. sedebokerense* 27 h after inoculation on *H. pluvialis* cells releasing amoeboid swarmers through the germ pore (arrow), visualized under bright field (**C**) or fluorescence (**D**) after staining with Nile red; propagules inside and outside the sporangium are yellow stained in these conditions. (**E**,**F**) *P. sedebokerense* cysts (marked with arrows) attached to senescent cells under phase contrast (**E**,**G**), which shows yellowish autofluorescence (**H**,**F**). Scale bar 10 µm.

In the three different inoculated monocultures, two types of cysts, the vegetative cyst and the resting cysts, were produced, and also both types of propagules, amoeboid swarmers and flagellated zoospores. Although these observations were not systematically quantified, we observed that resting sporangia (Figure 6A) were more abundant and appeared faster in *S. dimorphus* cultures at 48 h after inoculation (Figure 6B). In contrast, in *H. pluvialis* infected culture, vegetative cysts developing into mature sporangia releasing amoeboid swarmers (Figure 6C,D) are more common in the first hours or days after inoculation. In *S. dimorphus, P. sedebokerense* cysts were frequently attached to senescent cells which did not shows the red typical autofluorescence of the chlorophyll (Figure 6E–H).

## 4. Discussion

We carried out a comprehensive quantitative study of infections of microalgae by an aquatic true fungus, inoculating—for first time—with a known number of pure isolated propagules (Figure S1). Previous efforts carried out, by homogenization of fungal culture and inoculating with a known density [13], can only be considered as semi-quantitative, since propagules and cysts burst by homogenization, so that there was no real estimation of the infecting units. We also characterized here, for the first time, the chronology of infection stages (Figure 1) of *P. sedebokerense*, starting with pure propagules in Ps-Hp coculture. We found that the time needed to complete the cycle (new propagules release) is 16 h, which is significantly shorter than the period previously reported (24–30 h) to reach the sporangium stage in *H. pluvialis* culture [13]. This is also lower than the periods reported to complete the cycle in chytrid parasites of phytoplankton: 2 days [8] or 4–8 [9] in *Rhizophydium planktonicum,* 3 days in *Rhizosiphon crissum* [11] or 7–8 days in *Rhizophydium scenedesmi* [12]. The knowledge on the chronology of infection in *H. pluvialis* allowed us to design experiments to quantify and compare the kinetics of infection of the three commercially valuable microalgae: *H. pluvialis*, *C. zofingiensis* and *S. dimorphus*, establishing the sampling times in the first 48 h after inoculation.

We found prevalence values similar to the ones reported by Gutman et al. [20], with inoculated *H. pluvialis* and *C. zofingiensis* cultures reaching 100% of prevalence, and a *Scenedesmus* species reaching prevalence of 20%. However, in the current work, the experiment took 48 h with a parasite: host ratio of 1:1 at the onset of inoculation (Figure 4), and we used *S. dimorphus* (UTEX 1237), while in Gutman et al. [20], the experiment took 7 days; the inoculated ratio was not reported and the infected *Scenedesmus* species was *S. vacuolatus* (one specific strain). The average number of cysts, which *H. pluvialis* cells carried 3 h after inoculation, was 1; at the used inoculation ratio of 1:1, it implies that almost each propagule successfully encysted. For this reason, the prevalence and intensity in *H. pluvialis* remained constant until the second day, where the prevalence reached a value of 100% after the newly released propagules were attaching. The case of *C. zofingiensis* was different: the prevalence was moderate (10%) until the 8th hour, but sharply increased (to 75%) afterwards. These results imply a pulse of propagule release between 8 h and 12 h, and with an additional pulse to reach the 100% of prevalence occurring at 31 h after inoculation. The values of prevalence and intensity for *S. dimorphus* increased slowly during the first 12 h and reached a plateau of 20% prevalence and 0.4 of intensity; we therefore suggest that a propagule pulse did not occur. Since we compare host species with different cell sizes: *H. pluvialis* is 11 times and *S. dimorphus* is four times bigger than *C. zofingiensis*, we calculated the algal areal density to normalize the pressure suffered by the host cells per unit area. In this case, we found the highest cysts number per surface area in *C. zofingiensis* (5), intermediate in *H. pluvialis* (2.2) and low in *S. dimorphus* (0.1). We therefore suggest that the shorter *P. sedebokerense* life cycle found in *C. zofingiensis* (12 h) as compared to *H. pluvialis* (16 h) is due to full consumption of the host constituents in the smaller densely infected *C. zofingiensis* cells. This also explains why *C. zofingiensis* inoculated cultures collapse faster than *H. pluvialis* inoculated at the same ratio (data not shown). Why *S. dimorphus* reaches a prevalence plateau and promotes the development of *P. sedebokerense* resting cysts remains a mystery, but we can hypothesize that *S. dimorphus* is a suboptimal host. In studies of host range, it was found that parasites can infect suboptimal hosts and produce descendants that were only capable of infecting the original host [39]. The results presented in Table 2 further support those findings. In fact, *S. dimorphus* would be considered as reservoir host for *P. sedebokerense*, since it can be infected at a moderate density without suffering lethal epidemics, but promotes resting cyst formation (Figure 6A), which can germinate in the short term (Figure 6B) or resist unfavorable conditions [13,21]. According to our observations (Figure 6E–H), *S. dimorphus* cells targeted by the parasite were mainly senescent, which have yellowish chloroplast autofluorescence, in line with previous study’s pictures [21].

We investigated two parameters of the fitness starting with the same number of propagules inoculating 11:1 *H. pluvialis*, 1:1 *C. zofingiensis* and 4:1 *S. dimorphus* in different algal densities offering the same surface area, giving the same probability to the propagules to find a host (Figure 5). *P. sedebokerense* propagules infect *H. pluvialis* (39% survival, 8 h after inoculation) with greater success than *C. zofingiensis* or *S. dimorphus* (11% survival, 8 h after inoculation). These successful encystment events *in H. pluvialis* produced one magnitude order more propagules than *C. zofingiensis* and *S. dimorphus*; *C. zofingiensis* produced more propagules than *S. dimorphus*, but only in the first sampling time (22 h). Why does infection of *H. pluvialis* provide higher success *to P. sedebokerense* than infection of *C. zofingiensis*? We suggest that it is related to cell size (the lower production in *S. dimorphus* can be related to cell size but also to being a suboptimal host), since bigger host cells offer higher amounts of nutrients, as it was reported before that host cell size affects sporangium size and the number of spores produced [43].

When we investigated cross infection, the virulence of the different inocula could be explained by the number of propagules which are produced in each inoculated culture. That is, as more propagules are produced in Ps-Hp coculture than in Ps-Sd coculture, more intensive infection occurs resulting in higher virulence of Ps-Hp than Ps-Sd as an inoculum. But the susceptibility of each microalgal species shown in the cross-infection experiment seems to be explained by, or at least be related to, the preference of the *P. sedebokerense* propagules to each algal species. Since in the mixed culture, *P. sedebokerense* showed higher preference to the more sensitive species (*H. pluvialis*), the preference showed by the parasite could be interpreted as evolutionary adaption to the best source of food. For instance, *Rhizosiphon akinetum* evolved to only infect the akinetums of the phytoplanktonic cyanobacteria *Planktothrix*, presumably because infecting akinetes provides more energy than infecting regular cells [11].

We inoculated the three different algae with different inoculum types (Figure 2), in different algal densities, and different *P. sedebokerense* amounts, and we never observed *S. dimorphus* culture collapse, even if inoculating with a high number of *P. sedebokerense* cells and maintaining the culture for weeks. We observed partial flocculation of the *S. dimorphus* cultures and confirmed the partial infection by microscopic observations. These findings are in agreement with the findings of Gutman et al. [20] for another *Scenedesmus* strain but, contradicting those of Letcher et al. [21] who isolated *P. sedebokerense* from an outdoor collapsed culture of *S. dimorphus* strain used in the current study. According to our results, using *P. sedebokerense* AZ_ISR strain, mainly senescent cells of *S. dimorphus* were infected and culture collapse was never observed as reported by Letcher et al. [21] using *P. sedebokerense* strain FD16. The observed differences in virulence of *P. sedebokerense* against *S. dimorphus* in our work and Lechter et al.’s work can be attributed to being different parasite strains with possibly different host ranges or host preferences. However, since the culture crush reported by Letcher et al. [21] was only in outdoor culture, we can speculate that this crush of the algal culture was produced due to outdoors abiotic conditions which are different from our tested indoor conditions. In addition, since outdoor cultures are not axenic, we also can consider biotic factors, including another agent causing the culture crush as coinfection with *P. sedebokerense*. More tests with both strains, under the same conditions are needed before conclusion can be drawn up regarding this parasite virulence towards different algal hosts.

## 5. Conclusions

Our system offers a useful tool to compare infections in different microalgae cultures, and the inoculation ratio can be adjusted for the specific purpose (e.g., can be decreased to mimic natural conditions).

Among the three economically valuable microalgae tested, *P. sedebokerense* showed higher preference to *H. pluvialis* when the three microalgae are present, correlating with the higher susceptibility of *H. pluvialis* and the faster infection development.

The life cycle of *P. sedebokerense* is shorter than previously assumed and this needs to be considered when developing effective strategies to control microalgae fungal pests. Rapid isolation and/or treatment of contaminated cultures is essential.

The infective cycle in *C. zofingiensis* was shorter than in *H. pluvialis*; we therefor suggest that this is due to the smaller host cell size.

We consider *S. dimorphus* as a reservoir host of *P. sedebokerense* since is not collapsing after infection and promotes resting cyst formation. Reservoir hosts would be a threat to be considered by microalgal industry, since their cultures could show less symptoms and spread the parasitic pest.

## Figures and Tables

**Table 1:** Preference test in a mixed algae culture. We measured the number of encystment events after 9 h, in a mixed algae culture containing the three microalgae (in a 10 mL flask), offering the same algal surface area to the inoculated parasite propagules. Hp: *H. pluvialis*, Cz: *C. zofingiensis* and Sd: *S. dimorphus*.

	Algae, Cells/mL	Blastoclad, Propagules/mL	% of Infected Cells	Cysts/Host Cell	Encystment Events	Preference, Encystment Events/Propagules
Hp	25,000	230,000	96.3	4.90	120,000	52.1%
Cz	210,000	230,000	9.0	0.17	3570	1.6%
Sd	75,000	230,000	7.1 × 10^−15^	4.4 × 10^−16^	3.3 × 10^−11^	0.00%

**Table 2:** Cross infections and mixed infection tests. Table shows the susceptibility of the algal monocultures and the virulence of the inocula from the different algal cultures, previously inoculated with the same number of blastoclad propagules for 5 days. The table shows the time in hours needed to flocculate (F) and collapse (C) the algal culture, as indicated by a change of color from green to brown. Ps: *P. sedebokerense*, Hp: *H. pluvialis*, Cz: *C. zofingiensis*, Sd: *S. dimorphus*. Mixed Infection is an infected mixture of the three microalgae used at a density of *H. pluvialis*, 2.5 × 10^4^; *S. dimorphus*, 7.5 × 10^4^ and *C. zofingiensis*, 2.1 × 10^5^.

Algal Culture	Infected Co-Culture Used as Inoculum							
	*Ps-Hp*		*Ps-Cz*		*Ps-Sd*		*Ps*-Mixed Algae	
	F	C	F	C	F	C	F	C
*Hp*	24 h	48 h	48 h	60 h	48 h	60 h	24 h	48 h
*Cz*	48 h	60 h	168 h	No	168 h	No	96 h	168 h
*Sd*	No	No	No	No	No	No	No	No

## Data Availability

Data is contained within the article and supplementary material, data to build Figure 4 and Figure 5 are contained in Supplementary Table S3, the ITS sequence of the strain used in this study is available in GenBank as detailed in the Material and Methods section.

## Supplementary Materials

The following are available online at https://www.mdpi.com/2309-608X/7/2/100/s1, Figure S1: isolated propagules of *P. sedebokerense*, Table S1: Surface areas of microalgae, Table S2: Inoculation details, Table S3: Resume of Generalized Linear Model (GLM).
