# Supplementary material for: Paraphysoderma sedebokerense Infection in Three Economically Valuable Microalgae: Host Preference Correlates with Parasite Fitness
Source: J Fungi (Basel). 2021 Feb 1;7(2):100. doi: 10.3390/jof7020100 (PMC7912770; doi:10.3390/jof7020100)
Supplement: Supplementary file 1 [file jof-07-00100-s001.zip › supplementary/Supplementary Table S1.pdf]

	Diameter/axis (µm)	Surface area (μm^2)	Area ratio
Hp	18	1018	11.5
Cz	5.3	88	1
Sd	11.4 * 3	345	3.9

Supplementary Table S1 Measurements of axis, volume and surface areas of *H. pluvialis* (Hp), *C. zofingiensis* (Cz) and *S. dimorphus* (Sd).
