# Supplementary material for: Paraphysoderma sedebokerense Infection in Three Economically Valuable Microalgae: Host Preference Correlates with Parasite Fitness
Source: J Fungi (Basel). 2021 Feb 1;7(2):100. doi: 10.3390/jof7020100 (PMC7912770; doi:10.3390/jof7020100)
Supplement: Supplementary file 1 [file jof-07-00100-s001.zip › supplementary/Supplementary Table S2.pdf]

	Inoculum type	Inoculum quantity	Algal cells/mL
Virulence I<br><br>**Fig 2A and 2B**	*P. Sedebokerense* pure culture or microalgal infections	Volumetrically from 1:100 to 1:10	Hp: $2*10^5$-$2*10^6$<br>Cz: $2*10^6$- $8*10^7$<br>Sd: $2*10^6$- $8*10^7$
Virulence II<br>**Fig 2C**	Isolated propagules from<br>*P. Sedebokerense* pure culture or microalgal infections	Up to 1:1 ratio Or Volumetrically<br>From 1:100 to 1:20	Hp: $2*10^5$-$2*10^6$<br>Cz: $2*10^6$- $8*10^7$<br>Sd: $2*10^6$- $8*10^7$
Infection parameters: (Prevalence, Intensity, Areal Density) **Fig 3 A**, **Fig 4**	Isolated propagules from *P. Sedebokerense* pure culture	Ratio 1:1<br>(number of propagules equal to algal cells)	Hp $4*10^5$<br>Cz $4*10^6$<br>Sd 1 $*10^6$
Fitness Parameters: (Propagule survival and Propagule production)<br>**Fig 3B, Fig 5**	Isolated propagules from<br>*P. Sedebokerense* pure culture	$4*10^6$ propagules/mL,<br>Ratio: Ps-Hp 10:1<br>Ps-Sd 4:1<br>PS-Cz 1:1	Hp $4*10^5$<br>Cz $4*10^6$<br>Sd 1 $*10^6$
Mixed infection<br>**Table 1**	Isolated propagules from<br>*P. Sedebokerense* pure culture	$2.3*10^5$ propagules /mL	Hp $2.5*10^4$<br>Cz $2.1*10^5$<br>Sd 7.5 $*10^4$
Cross-Infection<br>**Table 2**	Infected cultures inoculated with isolated propagules from:<br>*P. Sedebokerense* pure culture (one repetition), microalgal infections (one repetition)	Volumetrically from 5do infected cultures.<br>With initial composition:<br>Hp $7.6*10^4$ + 2.3*104 props.<br>Cz $7*10^5$ + 2.3*104 props.<br>Sd 2.5 $*10^5$ + 2.3*104 props.	Hp$7.6*10^4$<br>Cz $7*10^5$<br>Sd 2.5 $*10^5$
