# Supplementary material for: Paraphysoderma sedebokerense Infection in Three Economically Valuable Microalgae: Host Preference Correlates with Parasite Fitness
Source: J Fungi (Basel). 2021 Feb 1;7(2):100. doi: 10.3390/jof7020100 (PMC7912770; doi:10.3390/jof7020100)
Supplement: Supplementary file 1 [file jof-07-00100-s001.zip › supplementary/Supplementary Table S3.pdf]

Supplementary Table 3. Resume of GLM (left boxes) including the mean, error and confidence interval and post hoc Fisher methods (right boxes) showing between which groups are the significant differences (indicated with an asterisk) of Prevalence (A), Intensity (B), Propagules survival (C) and propagules production (D) for each sampling point. P-Value for each parameter at each sampling point is indicated on top of each box. Hp: *H. pluvialis*, Cz: *C. zofingiensis*, Sd: *S. dimorphus*

A

Host	Mean	St. Error	Lower Limit	Upper Limit	Host	Significance	Difference	+/- Limits
Prevalence 3 Hours			P-Value 0.001			Fisher	Method	
Hp	56.67	5.48	43.27	70.07	Cz - Sd		6.33	18.95
Cz	10.73	5.48	-2.67	24.13	Cz - Hp	*	-45.93	18.95
Sd	4.40	5.48	-9.00	17.80	Hp - Sd	*	52.27	18.95
Prevalence 8 Hours			P-Value 0.0004			Fisher	Method	
Hp	61.67	4.69	50.19	73.14	Cz - Sd		6.27	16.23
Cz	15.70	4.69	4.23	27.17	Cz - Hp	*	-45.97	16.23
Sd	9.43	4.69	-2.04	20.91	Hp - Sd	*	52.23	16.23
Prevalence 12 Hours			P-Value 0.006			Fisher	Method	
Hp	61.10	8.16	41.14	81.06	Cz - Sd	*	57.33	28.23
Cz	74.50	8.16	54.54	94.46	Cz - Hp		13.40	28.23
Sd	17.17	8.16	-2.79	37.13	Hp - Sd	*	43.93	28.23
Prevalence 22 Hours			P-Value 0.006			Fisher	Method	
Hp	89.67	10.68	63.53	115.80	Cz - Sd	*	63.98	36.96
Cz	83.33	10.68	57.20	109.47	Cz - Hp		-6.33	36.96
Sd	19.35	10.68	-6.78	45.48	Hp - Sd	*	70.32	36.96
Prevalence 26 Hours			P-Value 0			Fisher	Method	

Hp	97.62	2.62	91.21	104.02		Cz - Sd	*	80.00	9.06
Cz	100.00	2.62	93.59	106.41		Cz - Hp		2.38	9.06
Sd	20.00	2.62	13.59	26.41		Hp - Sd	*	77.62	9.06
Prevalence 31 Hours			P-Value 0				Fisher	Method	
Hp	94.43	4.35	83.80	105.07		Cz - Sd	*	79.86	15.04
Cz	100.00	4.35	89.37	110.63		Cz - Hp		5.57	15.04
Sd	20.14	4.35	9.51	30.77		Hp - Sd	*	74.29	15.04
Prevalence 48 Hours			P-Value 0				Fisher	Method	
Hp	100.00	3.34	91.82	108.18		Cz - Sd	*	79.82	11.57
Cz	100.00	3.34	91.82	108.18		Cz - Hp		0.00	11.57
Sd	20.18	3.34	12.00	28.36		Hp - Sd	*	79.82	11.57

B

Host	Mean	St Error	Upper Limit	Lower Limit		Contrast	Sig.	Difference	+/- Limits
Intensity 3 HAI			P-value 0.0017				Fisher	Method	
Hp	1.14	0.11	0.92	1.36		Cz - Sd		0.14	0.22
Cz	0.18	0.07	0.05	0.31		Cz - Hp	*	-0.95	0.26
Sd	0.04	0.09	-0.13	0.22		Hp - Sd	*	1.09	0.28
Intensity 8 HAI			P-value 0.001				Fisher	Method	
Hp	1.15	0.11	0.94	1.36		Cz - Sd	*	0.23	0.17
Cz	0.33	0.06	0.20	0.45		Cz - Hp	*	-0.82	0.24
Sd	0.09	0.06	-0.01	0.20		Hp - Sd	*	1.05	0.23
Intensity 12 HAI			P-value 0.0068				Fisher	Method	
Hp	1.19	0.13	0.94	1.43		Cz - Sd	*	0.89	0.35
Cz	1.06	0.13	0.80	1.31		Cz - Hp		-0.13	0.36
Sd	0.17	0.12	-0.07	0.41		Hp - Sd	*	1.01	0.35
Intensity 22 HAI			P-value 0.0263				Fisher	Method	
Hp	2.81	0.25	2.30	3.31		Cz - Sd	*	1.53	1.12
Cz	1.72	0.49	0.75	2.69		Cz - Hp		-1.08	1.09
Sd	0.19	0.29	-0.38	0.76		Hp - Sd	*	2.61	0.76
Intensity 26 HAI			P-value 0.0004				Fisher	Method	
Hp	10.13	0.51	9.13	11.13		Cz - Sd	*	1.36	1.34
Cz	1.56	0.51	0.56	2.56		Cz - Hp	*	-8.57	1.42
Sd	0.20	0.45	-0.69	1.09		Hp - Sd	*	9.93	1.34

Intensity 31 HAI			P-value 0			Fisher	Method	
Hp	14.83	0.60	13.63	16.03	Cz - Sd	*	3.36	2.38
Cz	3.60	1.14	1.32	5.88	Cz - Hp	*	-11.23	2.57
Sd	0.24	0.34	-0.45	0.92	Hp - Sd	*	14.60	1.38
Intensity 48 HAI			P-value 0			Fisher	Method	
Hp	24.81	0.25	24.32	25.30	Cz - Sd	*	4.60	0.57
Cz	5.00	0.20	4.60	5.40	Cz - Hp	*	-19.81	0.63
Sd	0.40	0.20	0.00	0.80	Hp - Sd	*	24.41	0.63

C

Host	Mean	St. Error	Lower Limit	Upper Limit	Host	Sig.	Difference	+/- Limits
	Propagules survival 2 Hours			P-Value 0				
Hp	69.55	5.34	57.48	81.61	Cz - Sd		7.50	17.07
Cz	17.73	5.34	5.66	29.80	Cz - Hp	*	-51.82	17.07
Sd	10.23	5.34	-1.84	22.30	Hp - Sd	*	59.32	17.07
	Propagules survival 5 Hours			-Value 0.0024				
Hp	81.21	9.07	59.03	103.40	Cz - Sd		3.26	31.37
Cz	13.48	9.07	-8.70	35.67	Cz - Hp	*	-67.73	31.37
Sd	10.23	9.07	-11.96	32.41	Hp - Sd	*	70.98	31.37
	Propagules survival 8 Hours			P-Value 0.048				
Hp	38.64	6.94	21.65	55.62	Cz - Sd		-2.01	24.02
Cz	10.11	6.94	-6.87	27.10	Cz - Hp	*	-28.52	24.02
Sd	12.12	6.94	-4.87	29.11	Hp - Sd	*	26.52	24.02

D

Host	Mean	St. Error	Lower Limit	Upper Limit	Host	Sig.	Difference	+/- Limits
Propagules production 22 Hours				P-Value 0.0001				

Hp	142.8	11.2	117.4	168.1
**Cz**	**83.5**	**11.2**	**58.2**	**108.8**
Sd	24.3	11.2	-1.1	49.6

Cz - Sd	*	59.3	35.8
**Cz - Hp**	*****	**-59.3**	**35.8**
Hp - Sd	*	118.5	35.8

Propagules production 24 Hours P-Value 0.0054

Hp	341.7	19.1	129.5	223.2
Cz	53.5	19.1	33.2	126.8
Sd	27.3	19.1	-11.2	82.5

Cz - Sd		44.3	66.2
Cz - Hp	*	-96.3	66.2
Hp - Sd	*	140.7	66.2

Propagules production 28 Hours P-Value 0.0001

Hp	279.7	21.3	224.8	334.5
Cz	19.3	21.3	-35.5	74.2
Sd	14.0	26.1	-53.2	81.2

Cz - Sd		5.3	86.7
Cz - Hp	*	-260.3	77.6
Hp - Sd	*	265.7	86.7

Propagules production 31 Hours P-Value 0.0006

Hp	100.5	3.92641	88.004	112.996
Cz	11.0	3.92641	-1.495	23.4956
Sd	3.0	3.92641	-9.495	15.4956

			Hp - Sd
Cz - Sd		-8.0	17.67
Cz - Hp	*	89.5	17.67
Hp - Sd	*	97.5	17.67
