# Supplementary figures and images for: Paraphysoderma sedebokerense Infection in Three Economically Valuable Microalgae: Host Preference Correlates with Parasite Fitness
Source: J Fungi (Basel). 2021 Feb 1;7(2):100. doi: 10.3390/jof7020100 (PMC7912770; doi:10.3390/jof7020100)

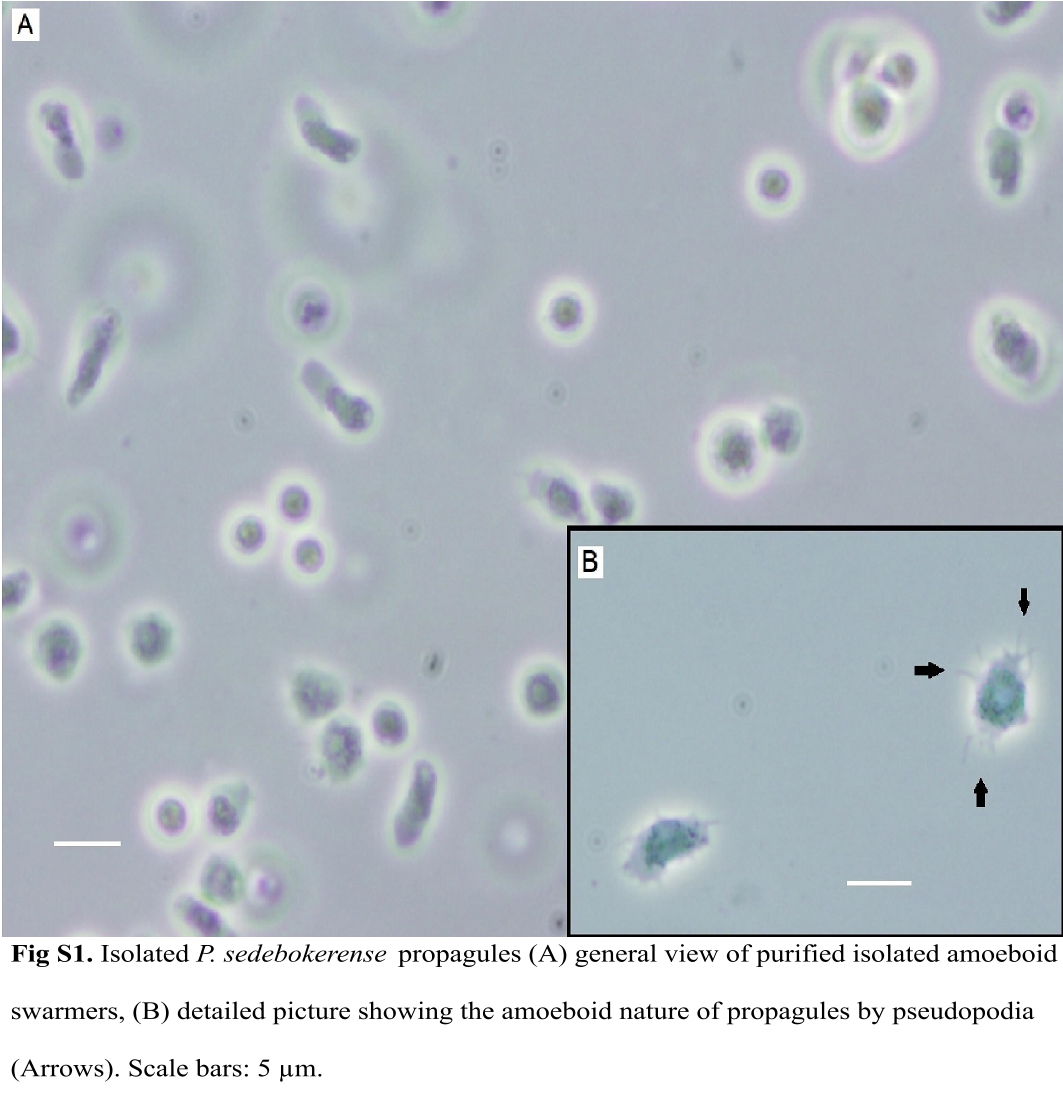

Supplement: Supplementary file 1 [file jof-07-00100-s001.zip › supplementary/FigS1.jpg]
